# Psychopathology after epilepsy surgery: a retrospective study

**DOI:** 10.1192/j.eurpsy.2022.1151

**Published:** 2022-09-01

**Authors:** C. Adão, D.F. Rodrigues, A.S. Sequeira, B. Silva, A. Velosa

**Affiliations:** 1 Centro Hospitalar de Lisboa Ocidental, Psychiatry Department, Lisboa, Portugal; 2 University of Birmingham, Clinical Neuropsychiatry, Birmingham, United Kingdom

**Keywords:** Psychopathology, epilepsy surgery, epilepsy

## Abstract

**Introduction:**

In patients submitted to refractory epilepsy surgery, psychiatric comorbidity is high (affecting 1 in every 3 patients), with descriptions of improvement, worsening and emergence of *de novo* psychopathology.

**Objectives:**

Identifying the prevalence of psychopathology and associated risk factors in a group of patients submitted to refractory epilepsy surgery.

**Methods:**

Retrospective observational study. Non systematic literature review.

**Results:**

We observed 42 patients, 45.2% female and 54.8% male, with an average age of 46.5 years (SD±11.6). The average age of presentation of epilepsy was 18.8 years (SD±12.7), 97.6% with temporal lobe epilepsy and 2.4% with parietal lobe epilepsy, 50% in each hemisphere. 19% had surgical complications and 40.5% had post-surgical recurrence of crisis. 45.2% presented with pre-surgical psychopathology (33.3% affective disorders, 16.7% anxiety disorders, 2.4% psychotic disorders, 2.4% neurodevelopmental disorders and 2.4% substance use disorders). Post-surgically, 50% improved, 20.8% maintained and 29.2% worsened their psychopathology and 21.4% had *de novo* psychopathology. We didn’t find associations between the analyzed variables and the worsening or appearance of *de novo* psychopathology.

**Conclusions:**

The worsening or appearance of *de novo* psychopathology is a well known phenomenon in patients submitted to refractory epilepsy surgery. In our sample there were cases of improvement, maintenance, worsening and emergence of *de novo* psychopathology, however we weren’t able to identify the factors associated with these different outcomes. Our study was retrospective and had a small sample, as limitations. Further, better-designed studies are necessary to identify risk factors for psychiatric disorders, allowing their effective prevention and treatment.

**Disclosure:**

No significant relationships.

